# Nativity, Racial/Ethnic, and Length of US Residence Differences in Chronic Kidney Disease: National Health and Nutrition Examination Survey 2011-March 2020

**DOI:** 10.21203/rs.3.rs-5760383/v1

**Published:** 2025-01-08

**Authors:** Oluwabunmi Ogungbe, Ruth-Alma Turkson-Ocran, Tosin Tomiwa, Khadijat Adeleye, Asma Rayani, Thomas Hinneh, Diana Baptiste, Melissa D. Hladek, Deidra C. Crews, Yvonne Commodore-Mensah

**Affiliations:** 1 Johns Hopkins University School of Nursing, Baltimore, Maryland.; 2 Johns Hopkins Bloomberg School of Public Health, Baltimore, Maryland.; 3 Beth Israel Deaconess Medical Center, Division of General Medicine, Boston, Massachusetts; 4 Harvard Medical School, Boston, Massachusetts.; 5 Elaine Marieb College of Nursing, University of Massachusetts Amherst, Massachusetts.; 6 Johns Hopkins School of Medicine, Baltimore, Maryland.; 7 Johns Hopkins Center for Health Equity, Johns Hopkins University, Baltimore, Maryland.

**Keywords:** Chronic Kidney Disease, Nativity, Immigrants, Healthcare Disparities, Acculturation, Ethnic Groups, Social Determinants of Health

## Abstract

**Rationale:**

The chronic kidney disease (CKD) burden in the US varies by race/ethnicity. It was unclear whether nativity status influences these disparities. This study compared CKD prevalence by nativity status, race and ethnicity, and length of US residence.

**Study Design:**

Cross-sectional analysis.

**Setting/Participants:**

We drew a weighted sample of 13,636 adults representing 155,147,141 Hispanic, White, Black, and Asian adults from the pooled 2011-March 2020 National Health and Nutrition Examination Survey (NHANES), which included 155,147,141 US- and foreign-born adults.

**Exposures:**

Nativity (US- or foreign-born), race/ethnicity, and length of US residence.

**Outcome:**

We defined CKD as eGFR <60mL/min/1.73m^2^ or a urinary albumin-to-creatinine ratio ≥30 mg/g.

**Analytical Approach:**

Survey-weighted multivariable Poisson models estimated associations among nativity status, race, and ethnicity, length of US residence, and CKD, adjusting for covariates.

**Results:**

The prevalence of CKD among US-born adults was 14.0%, vs. 11.5% of foreign-born. Foreign-born adults were less likely to have CKD (prevalence rate ratio [PRR]: 0.75, 95% CI 0.60–0.93) than US-born adults, adjusting for age, sex, and socioeconomic factors. Black adults were more likely to have CKD than White adults (PRR: 1.44, 95% CI 1.23–1.68); this difference was greater among US-born adults (PRR: 1.48, 95% CI 1.25–1.76). Among Hispanic and Asian adults, age- and sex-adjusted prevalence of CKD increased with longer length of residence in the US.

**Conclusions:**

There are clear CKD disparities related to nativity location and length of US residence, and these vary by race/ethnicity. Interventions addressing the unique challenges faced by populations most at risk for CKD, such as access to healthcare barriers and socioeconomic disparities, may help mitigate the burden of CKD and promote health equity.

## INTRODUCTION

Disparities in chronic kidney disease (CKD) prevalence and outcomes persist among historically marginalized racial/ethnic minority groups in the United States (US).^1,2^ The prevalence of CKD is higher in non-Hispanic Black adults (16%) compared to Hispanic (14%), non-Hispanic White (13%), or non-Hispanic Asian adults (12%).^1,3^ Persons from marginalized racial/ethnic groups are also more likely to progress from CKD to kidney failure than non-Hispanic White persons.^4^ While the impact of nativity (being foreign-born) on CKD prevalence and outcomes is less studied, research on other chronic diseases suggests foreign-born persons may have health advantages over their US-born counterparts.^5–7^ Lower CKD prevalence has been reported among foreign-born adults, varying by country/region of origin.^8^ Further research is warranted to clarify the relationship among nativity, race/ethnicity, and CKD outcomes in the US.

Black/African American and Hispanic persons decline faster in kidney function than White persons across numerous studies.^9,10^ This disparity results in a higher burden of CKD, cardiovascular disease (CVD), and other chronic disease complications among these marginalized racial/ethnic groups and among older adults and immigrants.^11,12^ Persons from these groups have a higher prevalence of comorbid conditions, including Type 2 diabetes and hypertension, which further can also accelerate kidney function decline. The complex interplay of sociodemographic, biological, and health access factors underlies disparities in kidney disease progression.

The burden of CKD among foreign-born adults in the US is unclear and may differ from native-born populations. With more than 47 million foreign-born adults living in the US, it is crucial to identify which factors influence CKD risk in this population.^12^ Social and structural inequities underlie many health disparities for immigrant groups, including language barriers, cultural beliefs, access to insurance coverage, lower income and educational attainment, exploitative working conditions, substandard living environments, and experiences of discrimination.^13,14^ These social determinants, directly and indirectly, increase the risk of CKD and progression to kidney failure for marginalized immigrant groups. However, resilience factors related to culture, family structure, diet, genetics, and acculturation may mitigate some harmful exposures.^15^ Understanding the vulnerabilities and strengths of immigrant subpopulations provides opportunities to screen, prevent, and manage CKD in culturally informed ways and to address root causes through policy and systemic reforms.^16^ This study examined differences in the prevalence of CKD in the United States by nativity status (foreign-born versus US-born) and race and ethnicity and examined the impact of length of US residence (a proxy measure of acculturation).

## METHODS

### Data Source and Study Population

We conducted a cross-sectional analysis using pooled 2011 to March 2020 National Health and Nutrition Examination Survey (NHANES) data.^17^ We combined data from 2011–2016 and 2017-March 2020. NHANES was suspended during the COVID-19 pandemic, and 2019–2020 data were collected only until March 2020. The CDC combined the 2017–2018 and 2019–2020 cycles to ensure nationally representative estimates. Participants 18 years or older with available data on serum creatinine measurements, nativity, race and ethnicity, and socioeconomic status were included for analysis (Figure S1). NHANES is approved by the research ethics boards of the National Center for Health Statistics. All participants provided written informed consent. Full details about the design and methodology of the NHANES are found elsewhere.^18^

### Exposures

**Figure 1.**
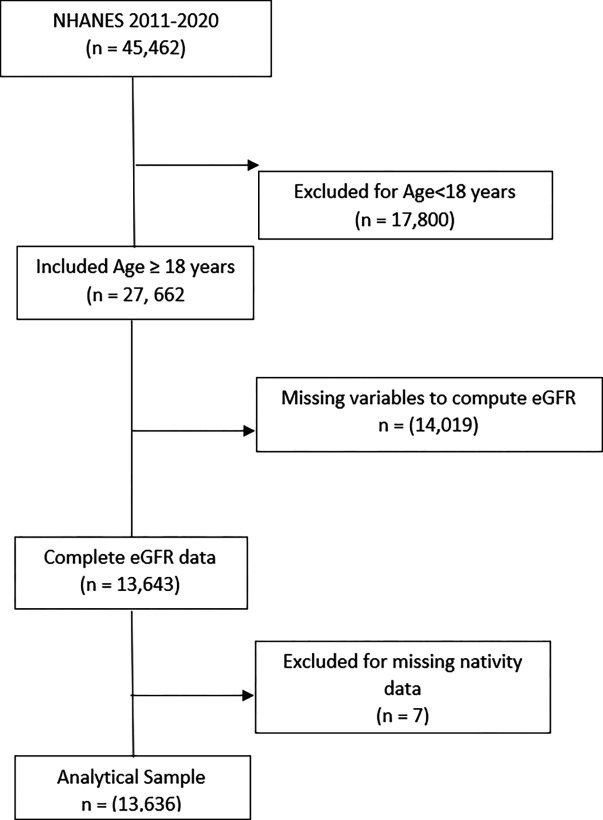
Sample Selection Outlining Inclusion and Exclusion Process

For nativity status, we dichotomized country of birth status into ‘US-born’ or ‘foreign-born.’ In the NHANES survey, participants were asked, “In what country were you born?” Participants’ responses were categorized as US-born (the 50 US states or Washington DC) or foreign-born. We used the year of US entry to calculate residence length, categorized as <5, 5 to <15 years, 15 to <30 years, 30 to <50 years, and ≥50 years. We also dichotomized at 10 years (<10 years and ≥10 years); this cut point is a commonly used indicator of acculturation among immigrants.^19^ Participants with missing nativity status data were excluded from the analysis (Figure 1). Race and ethnicity were combined into 4 groups: non-Hispanic White, non-Hispanic Black, Mexican American/Other Hispanic, and non-Hispanic Asian.

### Outcomes

The outcome of interest was the prevalence of CKD. We calculated the estimated glomerular filtration rate (eGFR) using the 2021 CKD-Epidemiology Collaboration (CKD-EPI) equation from calibrated serum creatinine measurements (eGFR 15–59 ml/min/1.73m^2^).^20,21^ We used the Kidney Disease: Improving Global Outcomes (KDIGO) definition of CKD, including persons with an eGFR <60 ml/min/1.73m^2^ or a one-time urine albumin-to-creatinine ratio ≥30 mg/g.^22^ Urinary albumin and creatinine were measured in a random urine collection as part of the medical examination of the NHANES examinations in the mobile examination clinics. The random urine albumin was initially measured in ug/mL, and urine creatinine was initially measured in mg/dL. Both measures were converted to the ACR in mg/g. We assessed urinary albumin excretion and categorized ACR into four categories: <10 mg/g, 10–29 mg/g, 30–300 mg/g, and >300 mg/g, based on the KDIGO guidelines.^23^

### Demographic Characteristics and Socioeconomic Factors

Sociodemographic characteristics included age in years (continuous), sex (female/male), health insurance (yes/no), employment status (yes/no), and smoking status (never/former/current). We also examined education, poverty income ratio (PIR), and body mass index (BMI) in categories. Education level was categorized into: ‘Did not complete High school,’ ‘High school/GED or equivalent,’ and ‘Some college and above’. BMI was calculated using respondents’ measured height and weight. Overweight and obese BMI categories were combined into one category, overweight/obese, and analyzed as a dichotomous variable vs. normal weight as the reference category. PIR was a proxy for income status and obtained by dividing the family income midpoint by the poverty threshold for each survey year. We categorized PIR into 4 levels: <1, 1–1.99, 2–3.99, and ≥4. A PIR <1 meant an individual was below the national poverty level. Participants were asked if they had “smoked at least 100 cigarettes in [their] entire life.” Those with an affirmative response were asked, “Do you now smoke cigarettes every day, some days, or none at all?” Based on this, we defined smoking as whether a person had smoked at least 100 cigarettes in their lifetime. Smoking status was categorized into never, former, or current smoker.

### Comorbidities

We examined the following variables as risk factors: diagnoses of hypertension, diabetes, and overweight/obesity. Hypertension was defined as systolic blood pressure ≥130 mmHg, diastolic blood pressure ≥80 mmHg, and current use of prescribed blood pressure medication as an affirmative answer to the question “Have you ever been told by a doctor or other health professional that you had hypertension, also called high blood pressure?” Participants were classified as diagnosed with Type 2 diabetes if they answered “yes” to the question: “Other than during pregnancy, have you ever been told by a doctor or health professional that you have diabetes or sugar diabetes?” Participants were classified with undiagnosed diabetes if they did not report a diagnosis of diabetes by a health care provider, but their fasting (8–24 hours) plasma glucose was ≥126 mg/dL or hemoglobin A1c was ≥6.5%. BMI was categorized as normal weight (18.5 – 24.9 kg/m^2^), overweight (25.0 – 29.9 kg/m^2)^, or obese (>30 kg/m^2^). Overweight and obese BMI categories were combined into one named overweight/obese and analyzed as a dichotomous variable with normal weight as the reference category.

### Statistical Analysis

NHANES has a complex sampling design, therefore, we pooled data from the 2011-March 2020 survey years to increase estimate-precision and weighted the survey using NCHS guidelines.^24^ Data analysis was performed using R^©,^ Programming and Stata^©,^/IC 16.1 (Stata Corps^©,^ College Station, Texas). We examined differences in sociodemographic characteristics between US- and foreign-born using survey-weighted t-tests and chi-square tests for continuous and categorical variables. We used survey-weighted generalized linear models with a Poisson distribution and a logarithmic link with linearized variance estimation to compare the prevalence of CKD by nativity and race and ethnicity in the US. We also fitted models that adjusted for age, sex, race and ethnicity, and age, sex, race and ethnicity, PIR, education, employment, and health insurance, respectively. We performed stratified analyses by nativity and separately by race and ethnicity. We fitted an interaction term for race, ethnicity, and nativity status and examined the margins. Finally, we examined length-of-US-residence trends in CKD prevalence, stratifying by race and ethnicity, and examined marginal-effect estimation.

Additionally, to further examine changes in estimates upon adjustments, we conducted a series of regression models with progressive adjustment for socioeconomic factors to examine how estimates for the associations of nativity and race/ethnicity with CKD prevalence changed upon accounting for these variables. Specifically, we fit models adjusting for: 1) age, sex, and race/ethnicity, 2) + PIR, 3) + educational attainment, 4) + employment status, and 5) + health insurance coverage. We also conducted mediation analyses to explore whether socioeconomic factors mediated the relationships between 1) nativity and CKD prevalence, and 2) race/ethnicity and CKD prevalence. Potential mediators examined included PIR, educational attainment, employment status, and health insurance coverage. We estimated the total, direct, and indirect effects using survey-weighted regression models

## RESULTS

We included a sample population of 13,636 representing 155,147,141 US- and foreign-born adults. Of these, 9,457 (69%) were US-born and 4,161 (31%) were foreign-born. The sociodemographic characteristics of the sample, stratified by nativity, appear in Table 1. The overall sample’s mean age (±SD) was 47.5(±15.8) years, and 52% were female. US adults more often had health insurance (89.7% vs. 73.2%, p<0.001) and a routine place for health care (85.0% vs. 75.8%, p<0.001), compared to foreign-born adults. The prevalence of hypertension (47.2% vs. 39.9%, p<0.01) and overweight/obesity (73.8% vs. 72.7%, p<0.001) was higher among US-born adults than foreign-born adults. The overall prevalence of CKD in the sample was 13.5% and was higher among US-born adults compared to foreign-born adults (14.0% vs. 11.5%, p<0.001). Among non-Hispanic White adults, those US-born had higher CKD prevalence than those foreign-born (13.5% vs. 8.5%, p=0.04). Similarly, among non-Hispanic Black persons, US-born adults had higher CKD prevalence than those who were foreign-born (19.2% vs. 9.5%, p<0.001, Table 2).

### Differences in CKD prevalence by nativity, race, and ethnicity

**Figure 2:**
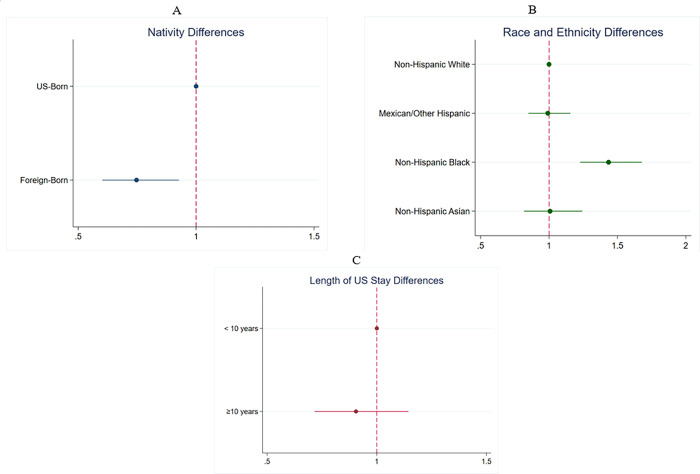
Adjusted Chronic Kidney Disease Prevalence Rate Ratio by A. Nativity, B. Race and ethnicity, and C. Length of US Residence. Plots show estimates from fully adjusted models; covariates were age, sex, race and ethnicity, poverty-income ratio, education, employment, and health insurance. Race/ethnicity was not included as a covariate in analyses for which race/ethnicity was the main exposure variable. PRR, Prevalence Rate Ratio, indicates the Prevalence Ratio derived from multivariable Poisson models. Results are weighted and adjusted for age, sex, race and ethnicity, poverty-income-ratio, education, employment, and health insurance. Estimates represent PRRs comparing each category to the reference group. Reference groups are US-born for nativity, non-Hispanic White for race/ethnicity, and <10 years for length of US residence. Bars represent 95% confidence intervals. Statistical significance (p<0.05) is denoted by error bars that do not cross the vertical line at PRR=1.

After we adjusted for age, sex, and sociodemographic characteristics (Table 3), foreign-born adults were 25% less likely to have CKD (prevalence rate ratio: 0.75, 95% CI 0.60–0.93) than U.S.-born adults (Figure 2). In the fully adjusted model, non-Hispanic Black adults were 44% more likely to have CKD than non-Hispanic White adults (PRR: 1.44, 95% CI 1.23–1.68). Also, in the fully adjusted model, among US-born adults only, non-Hispanic Black adults were 48% more likely to have CKD than non-Hispanic White adults (PRR: 1.48, 95% CI 1.25–1.76).

Among foreign-born adults, with age and sex adjustment, foreign-born Mexican/Other Hispanic adults had twice the odds (PRR: 2.00, 95% CI 1.22–3.30), and Asian adults had a 74% (PRR: 1.74, 95% CI 1.03–2.94) higher likelihood of CKD, compared to foreign-born White adults However, this was not significant in the fully adjusted model accounting for socioeconomic factors. Among non-Hispanic White adults, foreign-born adults had a 42% (PRR: 0.58, 95% CI 0.34–0.97) lower likelihood of CKD than US-born adults with age and sex adjustment only. In the fully adjusted model, foreign-born Black adults had a 51% (PRR: 0.49, 95% CI 0.33–0.74) lower likelihood of CKD compared to US-born Black adults. Although the likelihood of CKD was 63% (PRR: 1.63, 95% CI 1.33–1.98) higher among foreign-born adults who had lived in the US for ≥10 years, this effect was attenuated after adjustment for age, sex, and socioeconomic factors (Figure 2).

### Length of US residence trends of CKD by race and ethnicity

**Figure 3:**
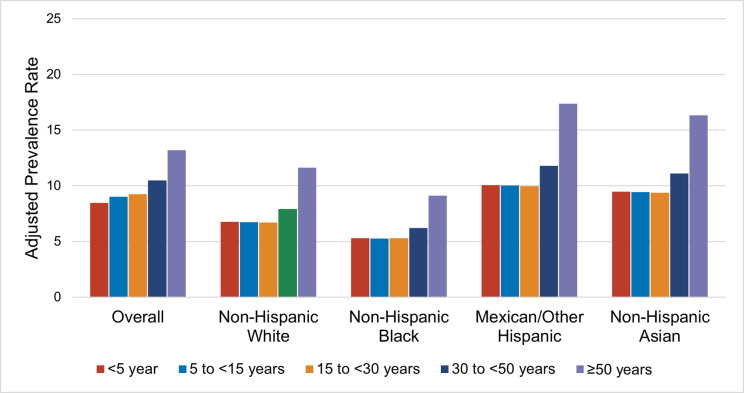
Age- and Sex-Adjusted Chronic Kidney Disease Prevalence Rate over Length of US Residence Among Foreign-born persons by Race and Ethnicity. Results are from marginal-effect estimates derived from survey-weighted generalized linear models with a Poisson distribution and log link, adjusted for age and sex. The y-axis represents the predicted prevalence (%) of chronic kidney disease (CKD) based on these models. The x-axis displays length of residence in the United States in years, categorized as <5, 5-<15, 15-<30, 30-<50, and ≥50 years. Results are stratified by race/ethnicity (Non-Hispanic White, Non-Hispanic Black, Mexican/Other Hispanic, and Non-Hispanic Asian).

There were temporal changes in CKD prevalence by length of residence in the US among foreign-born persons (Figure 3). Overall, the age and sex-adjusted prevalence of CKD increased with length of residence in the US, from 8.5% among persons who had lived <5 years in the US, to 13.2% among persons who had lived 50+ years in the US (p<0.001). CKD prevalence trends by length of US residence differed significantly by race and ethnicity. Among Mexican/other Hispanic persons, CKD prevalence increased from 10.1% among persons who had lived <5 years in the US to 17.4% among those who had spent 50+ years in the US. We observed a similar trend among non-Hispanic Asian persons (9.5% to 16.3%) and non-Hispanic White persons (6.8% to 11.6%). Although CKD prevalence was lowest across US residence years for non-Hispanic foreign-born Black persons, CKD prevalence increased over time, from 5.3% in those who had lived <5 years in the US, to 9.1 in those who lived ≥50 years in the US.

### Progressive Adjustments and Mediation Analyses

The association between nativity and CKD was minimally changed slightly after further adjusting for PIR, education, employment, and health insurance status (PRR 0.75, 95% CI 0.60–0.93) (**Table S1**). Relative to non-Hispanic White adults, CKD PRR for non-Hispanic Black adults was attenuated but remained significant (PRR 1.44, 95% CI 1.23–1.68) (**Table S2**). After further adjustment for PIR, the association became null for Mexican American/Other Hispanic adults (PRR 0.99, 95% CI 0.85–1.16), while non-Hispanic Black adults remained at higher risk though attenuated (PRR 1.43, 95% CI 1.23–1.67). Additional adjustments for education, employment, and health insurance status had minimal impact on these estimates (**Table S2**). The mediation analyses indicated that the association between nativity and lower CKD prevalence was partially mediated by higher PIR (≥2: PRR 0.77, 95% CI 0.65–0.91), greater educational attainment (≥college graduate: PRR 0.55, 95% CI 0.46–0.66), employment (PRR 0.39, 95% CI 0.34–0.44), and having health insurance (PRR 1.25, 95% CI 1.07–1.46), though the association remained after accounting for these factors (**Table S3**). Similarly, the association between race and ethnicity and CKD prevalence was partially mediated by these factors, with the effects being more pronounced among US-born adults compared to foreign-born adults (**Table S4**).

## DISCUSSION

Our study aimed to examine differences in CKD prevalence by nativity (US-born vs. foreign-born) and race and ethnicity in the US. We also investigated trends in CKD prevalence by length of US residence among immigrant subpopulations, including temporal patterns across race/ethnic groups.

Our analyses found: (1) Foreign-born persons were significantly less likely to have CKD than US-born adults. (2) There were significant racial/ethnic and nativity differences in CKD prevalence: non-Hispanic Black adults had a substantially higher CKD prevalence than non-Hispanic White adults, both in the overall sample and among US-born adults alone. Among non-Hispanic Black adults, foreign-born persons had a lower likelihood of CKD than those US-born. Among non-Hispanic White adults, foreign-born persons had a lower likelihood of CKD than those who were US-born. (3) There were differences in CKD prevalence by length of residence; foreign-born adults who had resided in the US for 10+ years had a higher prevalence of CKD than those who had been in the US for <10 years. Mexican and Other Hispanic persons were more likely to have a higher CKD prevalence across categories of length of residence in the US.

Our study extends an earlier study on CKD prevalence by nativity in the US,^8^ presenting findings on differences by nativity and length of US residence. Consistent with this prior study, we demonstrated that US-born adults have a higher CKD prevalence than foreign-born persons. Factors related to migration and acculturation may contribute to the differences in CKD by nativity status.^7^ Racial and ethnic inequities substantially impacted CKD risk and outcomes. Our results and similar findings show persistent gaps in CKD prevalence across racial/ethnic groups, nativity, and socioeconomic status persisted over the past decades.^3^ These disparities persist across all stages of the disease, with implications for preventive measures, screening criteria for high-risk individuals, and specialized care and treatment options for those experiencing kidney failure.^2^

Extensive research consistently reveals that racial and ethnic minority groups, along with individuals of lower socioeconomic status, face a disproportionately higher burden of progressive CKD.^25^ Contributing factors include disparities in risk factors like diabetes and hypertension, further widening the gap in CKD prevalence and outcomes among different populations.^26^ In addition, CKD awareness is poor; 9 in 10 adults in the US who have CKD are unaware,^27^ and significantly low ascertainment and non-disclosure of CKD exist in primary care.^28^ Clinical use of a race-based eGFR equation in CKD assessment, diagnosis, and management is another significant factor perpetuating and exacerbating unequal public health decision-making related to CKD.^29^

Several factors may contribute to the lower CKD prevalence among immigrants compared to native-born individuals, particularly among Black and Hispanic populations. The “healthy immigrant effect” has been offered as an explanation for these differences, suggesting that immigrants may initially exhibit better health outcomes than their host country-born counterparts, possibly due to the selection of healthier individuals during the migration process.^30,31^ However, this effect is not universally observed and may vary depending on the specific immigrant group, health outcome, and host country context.^32^ Some immigrant groups, such as refugees or those from low-income countries, may have poorer health upon arrival compared to host country-born populations.^33,34^ Additionally, cultural factors such as diet, social support, and health behaviors among immigrant communities may be protective against cardiometabolic conditions, including CKD, at least initially. For example, some immigrant groups may adhere to traditional diets rich in fruits, vegetables, and whole grains, which are associated with a lower risk of chronic diseases.^35^ Strong family and community ties within some immigrant communities may also provide social support and buffer against stress, which are important for good cardiometabolic health outcomes.^36^

However, acculturation to US lifestyles and environments may erode some of these health advantages over time. As immigrants adapt to US cultural norms and adopt more sedentary lifestyles, processed diets, and other unhealthy behaviors, their risk for chronic diseases like CKD may increase.^37^ This acculturation process may be particularly pronounced among second-generation immigrants growing up in the US.^38^

Socioeconomic factors play a role, as many immigrants face challenges such as language barriers, limited healthcare access, discrimination, and occupational hazards, which can negatively impact their health over time.^39–41^ The migration journey itself differs substantially across immigrant subgroups. Foreign-born Mexican/Other Hispanic adults had significantly lower educational attainment, income status, rates of health insurance coverage, and access to routine healthcare compared to their US-born counterparts. This shows the distinct migration journey experienced by many in the Hispanic population - reasons for migration like economic pressures or fleeing adverse circumstances, immigration status affecting eligibility for services, and geographic proximity to the US can lead to different socioeconomic trajectories.^42^ The complex interplay of acculturation stresses, evolving socioeconomic circumstances, and changing risk factor profiles likely contribute to the higher CKD prevalence observed across longer US residencies, specifically among Mexican/Other Hispanic persons.^43^ It is challenging to disentangle the influence of socioeconomic status from other cultural and behavioral factors impacting health outcomes in Latino populations.^44^ Our findings reinforce that any health advantages observed among immigrants may be temporary, contingent on the social, economic, and cultural contexts shaping their experiences in the US over time.

Our study found that length of residence in the US was an important factor in CKD prevalence. Persons who had lived longer in the US had a higher likelihood of CKD than those who had resided in the US for shorter periods. This finding corroborates with another NHANES analysis (data from 2001 to 2014) reporting that foreign-born individuals who had lived in the US for ≥15 years showed similar CKD levels as their US-born counterparts, while those with <15 years of US residence had a notably lower estimated eGFR.^8^ Consequently, this evidence validates the impact of the length of US residence on the CKD risk. This relationship between length of US residence and CKD may partially be explained by a similar relationship between length of US residence and CKD risk factors such as diabetes, hypertension, obesity, etc.^37^ Increase in CKD prevalence trends with increasing length of US residence was notably higher among Mexican and other Hispanic adults and non-Hispanic Asian adults. High rates of CKD have been documented among different Asian populations (Chinese, Malay, and Indian),^45^ and among Mexican American persons in the US.^3^

Further, higher socioeconomic status appeared to have a protective effect, buffering CKD prevalence among foreign-born adults. This suggests that social and economic advantages enabled by the migration process may initially confer health benefits.^15^ Over time, though, acculturation in the US environment likely erodes some of this socioeconomic resilience. These findings highlight the need to understand vulnerabilities and strengths when examining the intersections of immigrant identity and health.

It is imperative to address specific factors contributing to the decline in kidney function among US and foreign-born adults as they spend more years in the US. Access to healthcare plays a vital role in diagnosing and treating CKD. We found that health insurance coverage among US-born adults was higher (85.6%) than for foreign-born adults (64.6%), as was access to routine places for health care (86% vs. 75.4%). Foreign-born persons may have limited access to healthcare, which can influence the utilization of essential health services. Barriers such as navigating the intricacies of the US healthcare system, obtaining health insurance, financial constraints, language barriers, cultural differences, concerns about immigration status, and transportation issues may account for some of these differences in healthcare access.^46^ Consequently, these factors may contribute to previously observed lower healthcare utilization among immigrants and inadequate preventative screening, detection, and management of declining kidney function and CKD among immigrants.^47,48^

Migration is an important social determinant of health,^15^ so there is a need to review existing discriminatory health policies that exclude immigrants, minoritized groups, and persons who may have lower socioeconomic conditions from access to quality healthcare. It is essential to tailor interventions that promote health-seeking behavior and enhance kidney health among this population. It is also important to increase healthcare providers’ awareness of these disparities among foreign-born persons, including applying cultural humility and patient-centered care principles.^49^ To improve the kidney health of foreign-born US adults, policy recommendations should include disaggregating data on immigrants by their country of origin.^50^ This will provide valuable insights into ethnic differences and uncover the social, economic, and political factors that impact health outcomes. Additionally, there is a pressing need for enhanced community awareness of comorbidities (diabetes, hypertension) and the major risk factors associated with CKD. Increasing early detection and treatment of declining kidney function can mitigate the progression to CKD and end-stage kidney disease.

This study has several notable strengths. We analyzed nationally representative data on US adults, potentially allowing generalization to non-institutionalized US adults. The analysis incorporated combined survey data spanning 10.2 years (from 2011 to March 2020). It included essential biological data on eGFR, BMI, and ACR, all measured during the medical examination sessions of NHANES data collection.

However, we acknowledge this study’s limitations. While NHANES data are considered valid and reliable, the study’s cross-sectional nature restricts extrapolation to causal inferences. In clinical practice, a clinical diagnosis of CKD requires an eGFR<60ml/min/1.73^2^ with evidence of kidney injury for three months or more. However, adopting this diagnostic criterion in this analysis was not possible; hence, the most recent 2021 CKD-EPI formula without a Black race term was applied to establish CKD diagnosis.^20,21^ Further, although the length of US residence is a proxy measure of acculturation, it does not adequately reflect the complex impact of immigration and acculturation on the health of immigrants.

### Conclusion

We assessed CKD prevalence in the United States by nativity status, race and ethnicity, and temporal length of US residence trends over time in large nationally representative data (NHANES 2011-March 2020). We found significant differences in the prevalence of CKD between US-born adults and foreign-born adults: among racial/ethnic groups and by length of residence in the US. Our findings highlight the importance of culturally relevant public health and healthcare policy initiatives to address the burden of CKD in the US-born population and sub-populations of foreign-born adults with higher CKD burden. These policies and initiatives should focus on health promotion strategies to improve kidney health and enhance screening for early detection and effective management of CKD and its associated risk factors. By doing so, we can work toward reducing the prevalence of CKD among US-born individuals and foreign-born sub-populations and improve kidney health outcomes.

## Figures and Tables

**Table 1.** Sample Characteristics in the 2011-March 2020 National Health and Nutrition Examination Survey Data by Nativity

Characteristics	Total, No (%)	US Born, No (%)	Foreign Born, No (%)	*P value*
Weighted, N	155,147,141	126,078,359	29,068,782	
Unweighted, N	13,636	9,475	4,161	
Age (mean ±SD)	47.5 (15.8)	47.8 (14.9)	46.1 (18.2)	
Female,	7,026 (51.6)	4,844 (51.7)	2,182 (50.8)	0.32
Race and Ethnicity				<0.001*
Non-Hispanic White	4,654 (66.0)	4,454 (77.9)	200 (15.6)	
Non-Hispanic Black	3,188 (11.3)	2,871 (12.4)	317 (6.9)	
Mexican/Other Hispanic	3,570 (16.7)	1,411 (8.8)	2,159 (50.3)	
Non-Hispanic Asian	1,613 (5.9)	199 (0.9)	1,414 (27.2)	
Education				<0.001*
≤ High School	5,692 (36.5)	3,568 (33.58)	2,124 (49.1)	
Some College	4,107 (31.7)	3,313 (34.2)	794 (20.8)	
≥College graduate	3,213 (31.8)	2,077 (32.2)	1,136 (30.1)	
PIR				<0.001*
<1	2,496 (13.6)	1594 (11.4)	902 (24.0)	
1–1.99	3,211 (19.7)	2244 (18.4)	967 (25.9)	
2–3.99	3,219 (28.3)	2397 (29.2)	822 (24.3)	
≥4	3,035 (38.3)	2240 (41.0)	795 (25.8)	
Employed	7,525 (62.2)	5,046 (61.7)	2,479 (64.6)	0.06
Smoking Status				<0.001*
Never	8,102 (60.0)	5,098 (57.1)	3,004 (72.0)	
Former	2,616 (22.0)	1,949 (23.2)	667 (17.3)	
Current	2,421 (18.0)	2,029 (19.7)	392 (10.7)	
Insured	11,337 (86.6)	8,227 (89.7)	3,110 (73.2)	<0.001*
Healthcare access	11,285 (83.3)	8,040 (85.0)	3,245 (75.8)	<0.001*
Hypertension	2,591 (45.9)	1,780 (47.2)	811 (39.9)	<0.01*
Diabetes	2,342 (12.8)	1,557 (12.6)	785 (13.9)	0.09
Overweight/Obesity	5,999 (73.6)	4,345 (73.8)	1,645 (72.4)	0.24
CKD	2,332 (13.5)	1,748 (14.0)	584 (11.5)	<0.001*
Length of residence				
<10 y	--	--	1,912 (49.3)	
>10 y	--	--	2,064 (50.7)	

* Statistically significant; P, p-value; SD, standard deviation; PIR, Poverty-Income Ratio; CKD, Chronic Kidney Disease; y, years

**Table 2.** Sample Characteristics by Race and Ethnicity in the 2011-March 2020 National Health and Nutrition Examination Survey Data

Variable	Non-Hispanic White			Non-Hispanic Black			Mexican/Other Hispanic			Non-Hispanic Asian		
	US Born	Foreign Born	*P value*	US Born	Foreign Born	*P value*	US Born	Foreign Born	*P value*	US Born	Foreign Born	*P value*
Weighted, N	93,949,316	4,414,618		14,901,903	1,947,710		10,673,404	14,281,378		1,136,125	7,728,547	
Unweighted, N	4,454	200		2,871	317		1,411	2,159		199	1,414	
Age (mean ±SD)	49.7 (11.7)	51.8 (10.9)		45.0 (22.7)	44.1 (19.3)		37.9 (17.8)	44.3 (17.7)		31.4 (17.6)	46.9 (20.6)	
Female	2,215 (51.4)	104 (50.5)	0.82	1,521 (55.6)	167 (54.8)	0.79	762 (51.1)	1,139 (49.3)	0.35	95 (48.9)	738 (53.6)	0.19
Education			0.23			<0.001*			<0.001*			<0.003*
≤ High School	1,549 (31.0)	58 (28.4)		1,221 (43.6)	115 (37.6)		596 (43.9)	1,506 (68.9)		37 (20.0)	424 (29.0)	
Some College	1572 (33.2)	62 (28.0)		1,024 (36.4)	91 (28.2)		444 (35.7)	388 (19.4)		49 (28.0)	227 (16.7)	
≥College graduate	1,166 (35.8)	76 (43.6)		498 (20.0)	102 (34.2)		223 (20.4)	214 (11.8)		90 (52.0)	726 (54.3)	
PIR			0.62			0.67			<0.001*			0.51
<1	514 (8.0)	33 (11.1)		684 (25.6)	59 (23.1)		262 (18.9)	634 (34.6)		17 (9.9)	165 (12.8)	
1–1.99	1,082 (16.4)	43 (17.3)		666 (26.0)	68 (22.9)		330 (24.8)	584 (32.7)		28 (16.7)	254 (19.7)	
2–3.99	1,158 (29.0)	48 (26.0)		672 (28.9)	81 (32.0)		372 (26.6)	372 (22.0)		60 (32.0)	306 (25.6)	
≥4	1,336 (46.7)	57 (45.6)		440 (19.4)	59 (22.0)		297 (26.7)	174 (10.6)		71 (41.4)	488 (42.0)	
Employed	2,270 (61.7)	101 (61.0)	0.87	1,560 (60.3)	215 (69.3)	0.01*	803 (65.1)	1,237 (65.5)	0.89	138 (72.3)	878 (62.3)	0.003*
Smoking Status			0.13			<0.001*			0.01*			0.56
Never	2,221 (56.3)	106 (53.5)		1,598 (60.0)	270 (86.3)		883 (66.8)	1,457 (70.6)		159 (83.5)	1,123 (81.4)	
Former	1,104 (25.4)	58 (32.8)		466 (14.6)	28 (8.0)		247 (15.8)	411 (17.1)		20 (8.4)	161 (11.0)	
Current	885 (18.3)	28 (13.7)		731 (25.4)	14 (5.7)		231 (17.4)	228 (12.3)		16 (8.1)	113 (7.5)	
Insured	3,999 (91.8)	177 (87.8)	0.12	2,402 (82.5)	250 (79.2)	0.22	1,191 (82.6)	1,361 (57.9)	<0.001*	180 (93.5)	1,259 (89.7)	0.16
Healthcare access	3,867 (86.4)	170 (80.8)	0.14	2,465 (83.4)	261 (80.9)	0.44	1,135 (77.4)	1,610 (70.9)	0.001*	150 (75.3)	1,143 (80.0)	0.17
Hypertension	826 (47.4)	31 (35.8)	0.11	549 (55.2)	72 (47.2)	0.17	300 (35.3)	467 (38.6)	0.36	25 (39.6)	230 (43.4)	0.66
Diabetes	644 (11.8)	18 (1.9)	0.06	565 (16.6)	54 (15.2)	0.57	241 (13.2)	466 (15.4)	0.16	14 (5.5)	235 (15.2)	<0.001*
Overweight/Obesity	2,023 (73.0)	85 (69.4)	0.51	1,454 (77.3)	130 (74.1)	0.48	554 (78.8)	950 (82.1)	0.15	65 (50.0)	448 (53.8)	0.77
CKD	831 (13.7)	26 (8.5)	0.04*	614 (19.2)	38 (9.6)	<0.001*	192 (10.3)	327 (12.3)	0.09	17 (10.2)	181 (12.0)	0.48
Length of residence												
<10 y	--	66 (38.5)		--	161 (50.4)		--	845 (48.4)		--	807 (55.4)	
>10 y	--	130 (61.5)		--	150 (49.6)		--	1,161 (51.6)		--	587 (44.6)	

* Statistically significant; P, p-value; SD, standard deviation; PIR, Poverty-Income Ratio; CKD, Chronic Kidney Disease; y, years

**Table 3.** Unadjusted and Adjusted Associations between Nativity, Race and Ethnicity, Length of Residency in the US, and Chronic Kidney Disease

Nativity, Race and Ethnicity	Prevalence Rate Ratios (PRR) of CKD (95% Confidence Intervals)		
	Unadjusted PRR	PRR*	PRR^†^
Nativity			
*US-born*	1.00	1.00	1.00
*Foreign-born*	**0.82 (0.72–0.92)**	**0.74 (0.61–0.89)**	**0.75 (0.60–0.93)**
Race and ethnicity			
*Non-Hispanic White*	1.00	1.00	1.00
*Non-Hispanic Black*	**1.56 (1.13–1.56)**	**1.67 (1.46–1.91)**	**1.44 (1.23–1.68)**
*Mexican/Other Hispanic*	**0.85 (0.74–0.98)**	**1.25 (1.10–1.43)**	0.99 (0.85–1.16)
*Non-Hispanic Asian*	0.88 (0.71–1.07)	1.01 (0.91–1.32)	1.01 (0.82–1.24)
Length of Residence in the US			
*< 10 y*	1.00	1.00	1.00
*≥ 10 y*	**1.63 (1.33–1.98)**	0.91 (0.75–1.08)	0.91 (0.72–1.14)
**Stratified analyses by Nativity Status**			
US-Born Adults			
*Non-Hispanic White*	1.00	1.00	1.00
*Non-Hispanic Black*	**1.40 (1.19–1.65)**	**1.73 (1.51–2.00)**	**1.48 (1.25–1.76)**
*Mexican/Other Hispanic*	0.75 (0.61–0.92)	**1.27 (1.07–1.517)**	1.02 (0.82–1.26)
*Non-Hispanic Asian*	0.75 (0.40–1.40)	1.73 (0.88–3.35)	1.71 (0.86–3.39)
Foreign-Born			
*Non-Hispanic White*	1.00	1.00	1.00
*Non-Hispanic Black*	1.17 (0.70–1.98)	1.57 (0.87–2.86)	1.14 (0.64–2.05)
*Mexican/Other Hispanic*	1.47 (0.96–2.23)	**2.00 (1.22–3.30)**	1.40 (0.86–2.27)
*Non-Hispanic Asian*	1.43 (0.91–2.27)	**1.74 (1.03–2.94)**	1.41 (0.87–2.29)
**Stratified analyses by Race and Ethnicity**			
Non-Hispanic White			
*US-born*	1.00	1.00	1.00
*Foreign-born*	0.65 (0.41–1.01)	**0.58 (0.34–0.97)**	0.66 (0.39–1.11)
Non-Hispanic Black			
*US-born*	1.00	1.00	1.00
*Foreign-born*	**0.50 (0.35–0.73)**	**0.53 (0.37–0.76)**	**0.49 (0.33–0.74)**
Mexican/Other Hispanic			
*US-born*	1.00	1.00	1.00
*Foreign-born*	1.08 (0.92–1.26)	0.99 (0.81–1.23)	0.94 (0.75–1.17)
Non-Hispanic Asian			
*US-born*	1.00	1.00	1.00
*Foreign-born*	1.20 (0.97–1.49)	0.64 (0.35–1.15)	0.70 (0.37–1.32)

* Adjusted for age, sex, race and ethnicity

† Adjusted for age, sex, race and ethnicity, poverty-income-ratio, education, employment, and health insurance. Race/ethnicity was not included as a covariate in analyses stratified by race and ethnicity.

PRR, Prevalence Rate Ratio, indicates the Prevalence Ratio derived from the multivariable Poisson model. Results are weighted; CI, Confidence Interval; Ref, Reference; Bold - Statistical significance: *P<0.05*; y, years

## Data Availability

A detailed description of the NHANES survey is available at: http://www.cdc.gov/nchs/nhanes.htm. We used NHANES 2011–2020 data for our analysis. The National Health and Nutrition Examination Survey (NHANES) is a nationally representative survey conducted by the National Center for Health Statistics (NCHS). It employs a stratified, multistage probabilistic sampling design to provide national estimates of health indicators for the civilian, noninstitutionalized population of the United States. A detailed description of the survey methodology and data collection procedures is available in the NHANES analytic guidelines^14^
